# An overview of JAK/STAT pathways and JAK inhibition in alopecia areata

**DOI:** 10.3389/fimmu.2022.955035

**Published:** 2022-08-30

**Authors:** Maddison Lensing, Ali Jabbari

**Affiliations:** ^1^ Department of Dermatology, University of Iowa, Iowa City, IA, United States; ^2^ Interdisciplinary Graduate Program in Immunology, University of Iowa, Iowa City, IA, United States; ^3^ Iowa City Veterans Affairs (VA) Medical Center, Iowa City, IA, United States

**Keywords:** alopecia areata, JAK/STAT, JAK inhibition, cytokines, clinical trials

## Abstract

Alopecia Areata (AA) is a common autoimmune disease characterized by non-scarring hair loss ranging from patches on the scalp to complete hair loss involving the entire body. Disease onset is hypothesized to follow the collapse of immune privilege of the hair follicle, which results in an increase in self-peptide/MHC expression along the follicular epithelium. Hair loss is associated with infiltration of the hair follicle with putatively self-reactive T cells. This process is thought to skew the hair follicle microenvironment away from a typically homeostatic immune state towards one of active inflammation. This imbalance is mediated in part by the dominating presence of specific cytokines. While interferon-γ (IFNγ) has been identified as the key player in AA pathogenesis, many other cytokines have also been shown to play pivotal roles. Mechanistic studies in animal models have highlighted the contribution of common gamma chain (γ_c_) cytokines such as IL-2, IL-7, and IL-15 in augmenting disease. IFNγ and γ_c_ cytokines signal through pathways involving receptor activation of Janus kinases (JAKs) and signal transducers and activators of transcription (STATs). Based on these findings, JAK/STAT pathways have been targeted for the purposes of therapeutic intervention in the clinical setting. Case reports and series have described use of small molecule JAK inhibitors leading to hair regrowth among AA patients. Furthermore, emerging clinical trial results show great promise and position JAK inhibitors as a treatment strategy for patients with severe or recalcitrant disease. Demonstrated efficacy from large-scale clinical trials of the JAK inhibitor baricitinib led to the first-in-disease FDA-approved treatment for AA in June of 2022. This review aims to highlight the JAK/STAT signaling pathways of various cytokines involved in AA and how targeting those pathways may impact disease outcomes in both laboratory and clinical settings.

## Introduction

Alopecia Areata (AA) is a prevalent autoimmune disease that carries a lifetime risk of approximately 2% (1). AA is characterized by nonscarring hair loss that typically appears as small patches on the scalp. Severe cases of AA can result in complete hair loss of the scalp (alopecia totalis, AT) or complete loss of hair on the entire body (alopecia universalis, AU). While this disease can affect people of all ages, the mean age range of the first instance of hair loss is 25 to 37 years of age (1). The physical loss of hair in AA patients is often accompanied with increased psychological burden, which may manifest as depression, anxiety, and/or an impaired quality of life when compared to healthy individuals (2). The cause of AA is obscure but is believed to arise from a combination of environmental influences and genetic factors, such as variants within loci or certain genes implicated in autoimmunity and T cell signaling (3). There is no cure for AA, and off-label treatments, such as topical or locally-injected agents or systemic immunosuppressants, are commonly used, although historically these agents have been shown to have variable or poor efficacy. In the past decade, a new strategy has emerged for treating AA which acts to dampen ongoing inflammation by inhibition of Janus kinase (JAK) signaling. As of June 2022, the FDA has approved one JAK inhibitor for the treatment of AA, marking the first and currently only on-label treatment for adults with AA. JAKs are signaling mediators that function to convert the engagement of cytokine receptors with cognate cytokine to downstream effects. Many cytokine pathways that contribute to the pathogenesis of AA, such as interferon-γ (IFNγ) and those in the common gamma chain (γ_c_) family of cytokines, act by way of JAK signaling. Counteracting the effects of these cytokines with JAK inhibitors (JAKis) has shown efficacy in treating AA.

## Relevant pathophysiology of AA

**Figure 1:**
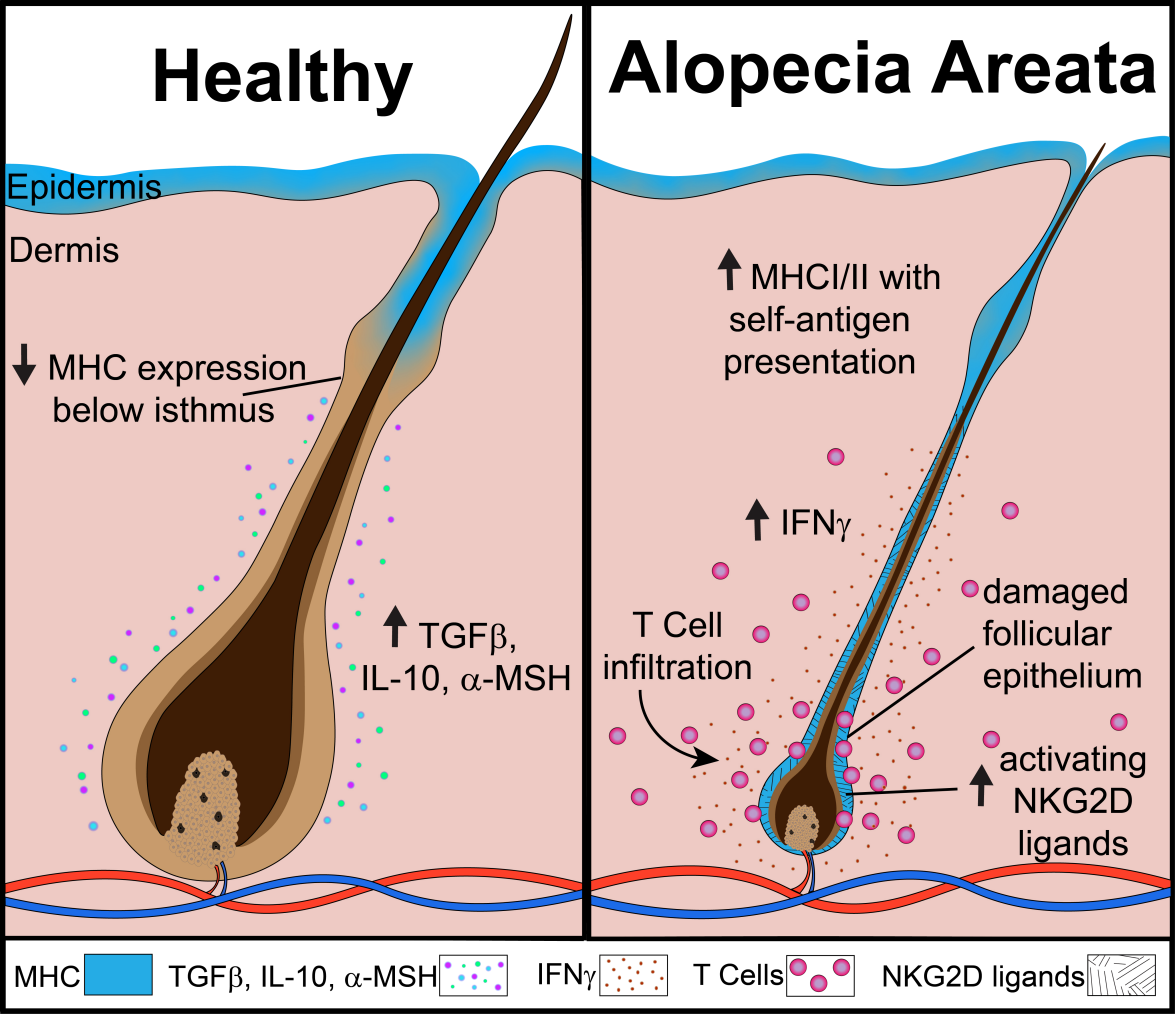
The collapse of immune privilege in the anagen hair follicle during alopecia areata.

AA is a disease that affects the hair follicles, which are known to follow a cyclic physiologic pattern. Broadly, there are three phases: anagen, a growth phase; catagen, a transition phase; and telogen, a rest phase. The anagen hair follicle is known to reside in a state of immune privilege (IP), which is characterized by low levels of MHC protein expression in its inferior portion, a relative lack of danger or stress ligands, the presence of anti-inflammatory cytokines/hormones (TGFβ, IL-10, α-MSH) and a paucity of immune cell infiltrates (4, 5). This IP state is thought to help protect the follicle from unwanted immune-mediated attack during this time. Maintaining this evasive state may be particularly important during the anagen phase, where a large number of tissue-specific peptides and antigens are generated. However, the microenvironment of the hair follicle exhibits dramatic changes in the AA disease state. In hair follicles in AA lesions, there is an increase in expression of MHC Class I and MHC Class II, upregulation of danger ligands (such as NKG2D activating ligands MICA and ULBP molecules), increased levels of proinflammatory cytokines, and a robust immune cell infiltrate (4, 6) (**Figure 1**). Immune cell infiltrates are comprised predominantly of CD4^+^ and CD8^+^ T cells. CD8^+^ T cells in close association with the hair follicle were found to express NKG2D, an activating receptor commonly associated with the natural killer (NK) cell lineage (6). This population of CD8^+^ NKG2D^+^ T cells was found to be sufficient for induction of disease in the cell-transfer murine model of AA (7). Activated CD8^+^ T cells are potent producers of pro-inflammatory cytokines, such as IFNγ, which skew the hair follicle microenvironment towards a proinflammatory state. Additionally, CD8^+^ T cell cytotoxic activity is known to be enhanced in the presence of cytokines such as those of the γ_c_ family. CD4^+^ T cells are also capable of producing cytokines that may contribute to the inflammatory state of the hair follicle during AA. Activated CD4^+^ T cells are known to produce the γ_c_ cytokine IL-2, which may enhance CD8^+^ T cell function. Additionally, a subset of CD4^+^ T cells known as T helper type 1 (Th1) cells produce IFNγ in an activated state, which may contribute to the inflammatory setting of the AA hair follicle. Substantial evidence demonstrates that a variety of potent cytokines and their respective signaling pathways are contributing to the pathogenesis of AA by driving functional and phenotypic changes in populations of T cells and in the hair follicle.

## JAK/STAT signaling

**Figure 2:**
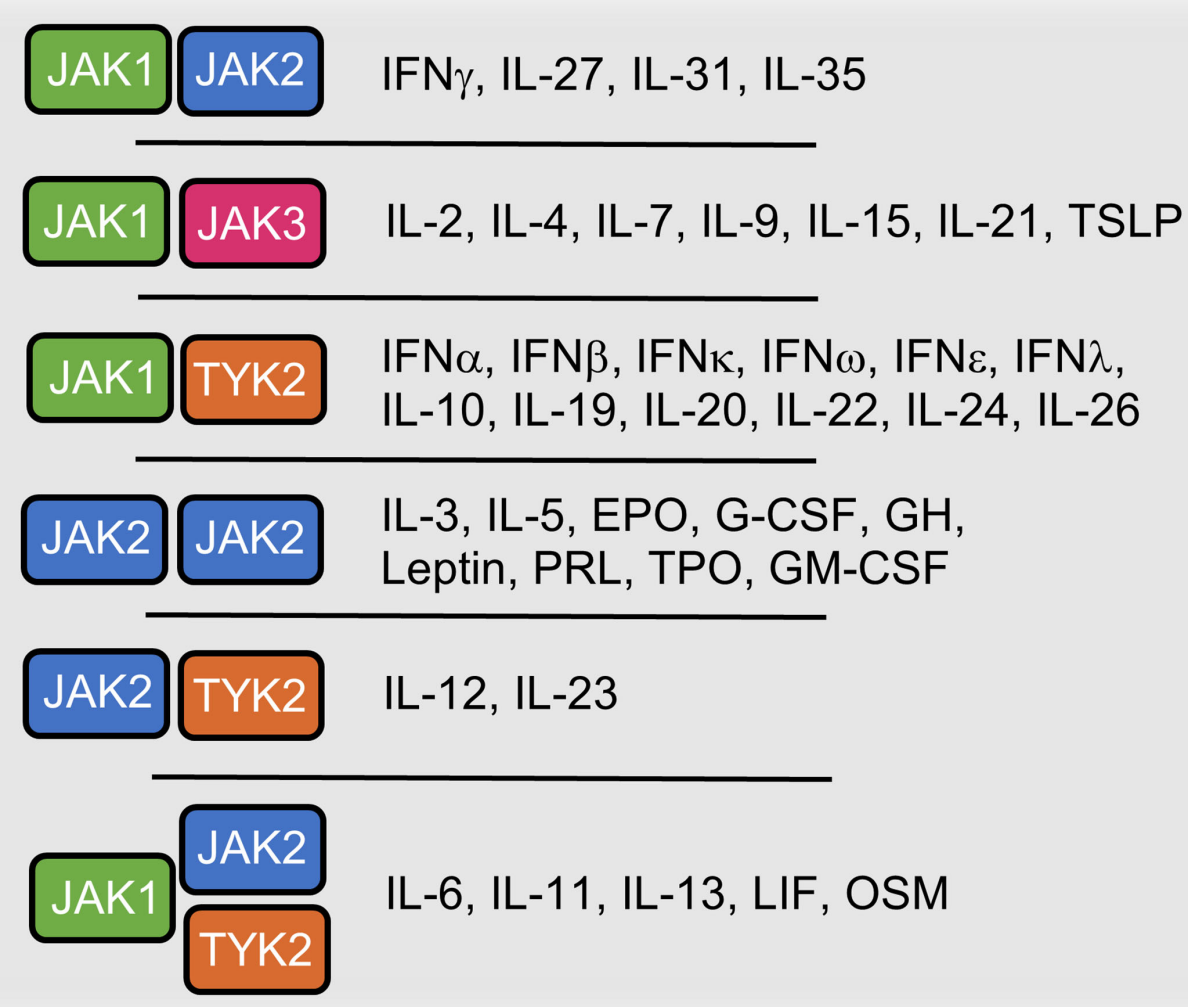
Cytokines and hormones that utilize JAK-mediated signaling. Interferon (IFN), Interleukin (IL), Thymic stromal lymphopoietin (TSLP), Erythropoietin (EPO), Granulocyte colony-stimulating factor (G-CSF), Growth Hormone (GH), Prolactin (PRO), Thrombopoietin (TPO), Granulocyte-macrophage colony-stimulating factor (GM-CSF), Leukemia inhibitory factor (LIF), Oncostatin M (OSM).

A large number of cytokine receptors lack intrinsic kinase activity, necessitating intermediaries, including tyrosine kinases, to transmit their downstream signals. By engaging their receptors, many types of growth factors and cytokines elicit their effects through use of the JAK molecules (**Figure 2**). There are four members of the JAK family of tyrosine kinases: JAK1, JAK2, JAK3, and TYK2. They are unique from other families of tyrosine kinases by virtue of their domain structure involving seven similar JAK Homology (JH) domains. All members contain a kinase domain (JH1) at their C-terminus region, and the phosphorylation of a conserved tyrosine residue within this region stimulates their catalytic activity (8). At their N-terminal region, JAKs contain a Band-4.1, ezrin, radixin, moesin (FERM) domain that mediates their interactions with cytokine receptors (9). Inactive JAKs may be found uncomplexed in the cytosol or pre-associated *via* their membrane-proximal cytoplasmic region of cytokine receptor subunits at their Box1 and Box2 motifs. The sequence of these two motifs determines with which cytokine receptor the JAK molecule will interact (10). Cytokine ligation results in conformational changes in the receptor that lead to dimerization of receptor subunits and in the juxtapositioning of the pre-associated JAKs or the recruitment of free JAKs to their docking sites. This proximity allows for the auto- or trans-phosphorylation of conserved tyrosine (Tyr) residues of the kinase domain and subsequent activation of the JAKs. The activated JAKs then go on to phosphorylate Tyr residues found on the distal intracellular region of the cytokine receptor, creating a docking site for the Src homology-2(SH2) domains of recruited signal transducers and activators of transcription (STATs).

There are seven different members of the STAT family, STAT1, STAT2, STAT3, STAT4, STAT5a, STAT5b, and STAT6. They share six similar domains related to their function. All STATs contain a centrally located DNA-binding domain, which allows for recognition of, and binding to, DNA sequences of target genes. This is followed by the SH2 domain, which can recognize tyrosine-phosphorylated (p-Tyr) sites on cytokine receptor subunits to facilitate STAT docking; each cytokine receptor subunit contains different STAT binding motifs, and the sequence surrounding the p-Tyr dictates which STAT will bind with highest affinity (10). At the C-termini of STATs are the transcription activation domain (TAD), which contains serine phosphorylation sites that direct transcriptional activity and contain binding sites for nuclear cofactors (11). Upon their recruitment to docking sites on cytokine receptors, STATs are phosphorylated by JAKs at a tyrosine residue located between the SH2 domain and TAD. This phosphorylation results in the SH2 domain of each STAT releasing from the receptor subunit and instead binding to the p-Tyr site on the other STAT monomer, resulting in the formation of a STAT dimer in active conformation with exposed DNA-binding sites. Dimers then translocate into the nucleus and bind to target genes within the DNA that possess STAT-binding sites, which ultimately results in inflammatory gene transcription, and the production of pro-inflammatory cytokines (10).

## Cytokine signaling in AA

Pro-inflammatory cytokines have been shown to play key roles in the pathogenesis of AA. Mechanistic studies highlighting the importance of these cytokines in disease onset and development have been performed using murine models of alopecia. The C3H/HeJ (referred to C3H herein) strain of mice spontaneously develops hair loss that recapitulates many features of human AA. In particular, C3H AA is a non-scarring form of alopecia, exhibits waxing and waning cycles, and is marked by lymphocytic infiltrates around the hair follicle, including CD8^+^ and CD4^+^ T cells (12). Additionally, previously unaffected mice can be induced to develop AA in a reproducible manner by using two different methods. One method consists of grafting a piece of skin from an alopecic mouse, e.g. a mouse that spontaneously developed AA, onto the dorsal region of a naïve recipient mouse (13). Another method involves collecting the skin-draining lymph nodes from a previously-affected mouse, activating and expanding the T cell populations *in vitro*, and then injecting the resulting cell population intra-dermally into naïve recipient mice (14). The development of these induction methods, resulting in tractable murine AA models that rapidly develop disease in a reproducible manner, have made it feasible to study and unravel the pathogenesis of this disease and has furthered our knowledge obtained from human AA patients, which had been largely limited to observational or *in vitro* studies. In particular, the roles of IFNγ and γ_c_ family members, including IL-2, IL-7, and IL-15, have been specifically investigated. The findings from these studies will be discussed in more detail below.

### Interferon-γ (IFNγ)

IFNγ is a 17 kDa protein that is biologically active as a homodimer; it is produced at high levels by activated CD4^+^ T helper type 1 (Th1) and CD8^+^ T cells, γδ T cells, and NK cells, and to a lesser extent by NKT cells, B cells, and antigen-presenting cells (15). The secretion of IFNγ is often associated with host defense towards foreign pathogens and is known to aid in tumor surveillance and the anti-tumor response. IFNγ is the sole member of the Type II IFN class of cytokines and signals through a tetrameric receptor comprised of two IFNGR1 chains and two IFNGR2 chains. Nearly all types of mammalian cells express the IFNγ receptor and are thus responsive to the effects of IFNγ to some extent. The level of IFNγ sensitivity is largely controlled by the level of expression of the IFNGR2 chain (16). Typically, IFNGR1 is readily expressed at relatively moderate levels, while the IFNGR2 chain can be upregulated from its low-level baseline expression during cases of activation or based on cell differentiation status (15). IFNGR1 chains have binding sites for JAK1, and IFNGR2 chains have binding sites for JAK2. Cytokine recognition by the IFN-γ receptor leads to autophosphorylation of JAK2 proteins; these active JAK2 molecules then go onto trans-phosphorylate JAK1 proteins. Activated JAK1 molecules each phosphorylate a tyrosine residue on their respective IFNGR1 chain, leading to the dual recruitment and activation of STAT1 monomers (16). Active STAT1 homodimers, also known as gamma-activated factors (GAFs), translocate to the nucleus and bind to the gamma-activated sequence (GAS) to drive target gene transcription (17).

In the setting of AA, sera from patients contain significantly higher levels of IFNγ when compared to healthy patients (18–20). IFNγ is hypothesized to play an important role in the collapse of the immune privileged state of the hair follicle. The ability of IFNγ to cause IP collapse has been demonstrated in experiments where anagen-stage hair follicles collected from a healthy scalp were cultured in the presence of IFNγ, resulting in increased expression of MHC I molecules (21). In a murine skin-graft induction model of AA, IFNγ has been shown to play a critical role in disease onset, as IFNγ-deficient mice fail to develop disease after grafting (22). Mice treated with an IFNγ neutralizing antibody starting at the time of grafting were prevented from developing disease, had a reduction in skin-infiltrating CD8^+^ NKG2D^+^ T cells, and failed to upregulate MHC I and MHC II molecules in the skin (7). Interestingly, groups have shown that intradermal injection of IFNγ alone or in combination with poly[I:C] into anagen-induced skin of naïve mice can drive the emergence of AA (23, 24). Overall, IFNγ has been identified as one of the main cytokines contributing to disease and does so, at least in part, by driving changes in the follicular epithelium to disrupt hair follicle immune privilege.

### Interleukin-2 (IL-2)

IL-2 is a 15 kDa protein that is mainly produced by activated CD4^+^ T cells and, to a lesser extent, by activated CD8^+^ T cells and DCs, NK cells, and NKT cells (25). IL-2 can signal through a high-affinity trimeric receptor (K_d_ ~10^-11^ M) or an intermediate-affinity dimeric receptor (K_d_ ~10^-9^ M) (26). The trimeric receptor consists of a unique IL-2Rα (CD25) subunit, the shared IL-2/15Rβ (CD122) subunit, and the γ_c_ (CD132) subunit, while the dimeric receptor consists of only the β and γ_c_ subunits. In order to form the trimeric receptor, IL-2 first weakly binds to IL-2Rα (K_d_ ~10^-8^ M); this initial interaction causes a conformational change in IL-2 that allows for association with the β subunit, recruitment of the γ_c_ subunit, and leads to the formation of a stable IL-2/receptor complex (27). Expression of the high-affinity, trimeric IL-2R is observed in CD4^+^ FoxP3^+^ regulatory T cells (Tregs) and activated conventional CD4^+^ or CD8^+^ T cells, suggesting that these populations of cells are exquisitely responsive to the effects of IL-2. The intermediate-affinity, dimeric IL-2R can be found on CD8^+^ memory T cells and NK cells, indicating they may also respond to IL-2. The dimeric receptor can also engage IL-15, and this promiscuity results in competition of the two cytokines for this receptor.

IL-2Rα is not known to engage any intracellular signaling molecules, whereas the IL-2Rβ subunit engages JAK1, and the γ_c_ subunit engages JAK3. Activation of JAK1/3 leads to subsequent activation and formation of STAT5/5 or STAT3/5 dimers that translocate to the nucleus to drive gene transcription programs that promote cellular expansion, survival, and maintenance. Additionally, STAT5 plays an important role in influencing T cell lineage fate. STAT5 is known to drive the expression of the Th1/Tc1 fate determining transcription factor T-bet which is crucial for acquisition of effector phenotype and functions, such as inducing CXCR3 expression and IFNγ production (28). There is also a STAT5 binding site in the CNS2 region of the *FOXP3* gene, which implicates STAT5 as a stabilizer of Foxp3 expression, which is important for the potent immunosuppressive phenotype and function of Tregs (29). Thus, IL-2 is a pleotropic cytokine that can enhance both effector and regulatory T cell populations that may work against each other in the context of an autoimmune disease such as AA.

In AA patients, increased mRNA expression of IL-2 is detected in the deep dermis of the skin, which contains the bulb of the hair follicle, when compared to levels of the upper dermis (30). Furthermore, active lesions of AA patients contain higher amounts of IL-2 mRNA than inactive areas, suggesting that IL-2 may be contributing to the localized areas of disease. A GWAS of AA patients and healthy controls revealed disease associations with SNPs in both the IL-2 gene and the IL-2Rα (CD25) gene (31). Various groups have profiled the serum of AA patients and observed higher levels of IL-2 in circulation in comparison to healthy patients (19, 20, 32). The crucial role of IL-2 has also been demonstrated with the murine skin-graft induction model. Graft-mediated induction experiments with IL-2^+/-^ mice, which possess only one functional IL-2 allele and produce 50% of typical IL-2 amounts, revealed that these mice were partially resistant to disease induction and had reduced leukocyte infiltration into skin lesions (33). Other reports demonstrated that mice treated with an anti-IL-2 neutralizing antibody at the time of grafting failed to develop disease, had a marked reduction of pathogenic CD8^+^ NKG2D^+^ T cells infiltrating the skin, and prevented the upregulation of MHC Class I and Class II in skin (7). Overall, IL-2 plays an essential function in T cell proliferation and homeostasis. The important roles of IL-2 in AA outlined above provides growing support for the role of γ_c_ cytokine family members in this disease and its relevant animal models.

### Interleukin-7 (IL-7)

IL-7 is a 17 kDa protein that is produced by various non-hematopoietic cells, such as stromal cells in the lymphoid organs or epithelial cells in the thymus or intestine (34). IL-7 signals through a dimeric receptor consisting of a unique IL-7Rα (CD127) subunit and the γ_c_ subunit. Expression of the IL-7 receptor can be found on naïve and memory αβ T Cells and is crucial for their development, survival, proliferation, and overall homeostasis. The IL-7Rα subunit preferentially binds JAK1, while the γ_c_ subunit binds JAK3. Activation of JAK1/3 leads to the recruitment and activation of STAT5, prompting the formation of a STAT5 homodimer that activates target genes (35). Genes transcribed after IL-7 signaling include the anti-apoptotic gene, *BCL2*, and genes that promote cell-cycle progression and differentiation, such as *MYC* and *PIM1* (36).

In AA patients, T cells infiltrating the HF were shown to have increased expression of IL-7Rα in comparison to T cells in control scalp samples (37). Furthermore, PBMCs isolated from AA patients stimulated in the presence of IL-7 resulted in significantly higher IFNγ production by CD8^+^ T cells in comparison to control patients (37). GWAS data has revealed one SNP in the IL-7Rα region associated with AA patients (31, 37). Together this suggests that T cells in both the circulation and skin of AA patients are more responsive to IL-7, and this may enhance their effector phenotype that promotes ongoing disease in these patients.

Murine studies of alopecia have demonstrated similar findings regarding the role of IL-7 and have also begun to unravel mechanisms of action. Gene expression analysis has revealed higher levels of IL-7 in AA skin when compared to skin of unaffected (UA) mice (37). The increase in IL-7 is likely due to increased production by keratinocytes, as they are known to upregulate IL-7 expression in the presence of IFNγ, which is likely released by CD8^+^ T lymphocytes infiltrating alopecic skin (37, 38). This suggests that a feedback loop exists between IL-7 production and IFNγ production of resident and infiltrating immune cells within the skin that may enhance the local pro-inflammatory environment. Mice treated with exogenous IL-7 complexed to an anti-IL-7 antibody, which expands the lifespan of IL-7 *in vivo*, experienced significantly quicker disease onset than mice receiving isotype control in a skin-graft induction setting. Additionally, mice treated with an anti-IL-7Rα blocking antibody at the time of skin-graft induction were completely resistant to development of disease. Clinical relevance of IL-7 blockade was demonstrated by the treatment of mice with anti-IL-7Rα after the first instance of hair loss. This treatment led to hair-regrowth in 60% of mice and reduced the inflammatory profiles of skin (37). While IL-7 is one of the more recent cytokines shown to contribute to the pathogenesis of AA, it again highlights the pertinent involvement of γ_c_ family members in enhancing this disease.

### Interleukin-15 (IL-15)

IL-15 is a 13kDa protein that is structurally similar to IL-2 and is produced by a variety of cells types, ranging from innate cells such as monocytes or dendritic cells to epithelial cells of the kidney, lung, heart, muscle and skin (39). IL-15 typically signals through a heterotrimeric receptor that consists of a unique IL-15Rα (CD215) subunit, the shared IL-2/15Rβ subunit, and the γ_c_ subunit. The IL-15Rα is expressed by lymphoid cells (including CD8^+^ T lymphocytes, NK Cells and γδ T lymphocytes), myeloid cells (including monocytes, macrophages, and dendritic cells), and nonhematopoietic cells (including colonic epithelial cells, microglial cells, or keratinocytes) (40). As discussed earlier (in the section on IL-2), expression of the β/γ_c_ subunits are often restricted to T lymphocytes or NK cells. IL-15 binds with highest affinity to the IL-15Rα (K_d_ ~ 10^-11^ M), which can then associate with the remaining β/γ_c_ subunits that are expressed on the same cell, known as cis-presentation (40). Interestingly, trans-presentation can also occur, in which a cell expressing the IL-15Rα subunit binds IL-15 and then presents it to another nearby cell expressing only the β and γ_c_ subunits to initiate IL-15-mediated signaling of the recipient cell. Of note, trans-presentation does not occur with IL-2, likely due to the lower binding of affinity of IL-2 with IL-2Rα (27). The IL-15/IL-15Rα complex has been shown to undergo endosomal recycling in certain cells, such as monocytes, which is a distinguishing feature from IL-2 (41). The high-affinity complex for IL-15 signaling involves engagement of all three (α/β/γ_c_) subunits. However, IL-15 can also signal through an intermediate-affinity complex consisting of only the latter IL-2/15Rβ and γ_c_ subunits (K_d_ ~ 10^-9^ M); as mentioned earlier, this results in competition of this receptor complex with IL-2 due to the redundant subunits (40). While the IL-15Rα subunit is not known to engage any JAKs, the remaining two subunits make use of JAK/STAT signaling networks to influence cellular actions. The shared IL-2/15Rβ subunit binds JAK1 leading to STAT3 or STAT5 activation, while the γ_c_ subunit binds JAK3 and leads to STAT5 activation. The STAT3/5 heterodimers or STAT5 homodimers lead to transcription of the anti-apoptotic gene *BCL2* and the proto-oncogenes *MYC*, *FOS*, and *JUN*, among others (42). IL-15 signaling is known to support CD8^+^ memory T cell survival, drive the expansion and maintenance of T cells and NK cells, and also enhance CD8^+^ T cell production of granzymes, IFNγ, and tumor necrosis factor α (42).

AA patients demonstrated increased levels of serum IL-15 compared to healthy controls, and patients with more severe forms of AA (AT or AU) exhibited significantly more IL-15 in comparison to patients with a few patches of hair loss (43, 44). Additionally, SALT scores, an AA clinical scoring system based on percentage of hair loss on the scalp, positively correlated with levels of circulating IL-15 (45). IL-15 levels in the serum have also been observed to correlate with disease duration (46). In comparison to healthy controls, both AA patients and AA mice have increased expression of IL-15 and IL-15Rα in the outer root sheath of the hair follicle (7). It is hypothesized that expression of the IL-15/IL-15Rα complex by skin cells engages the β/γ subunits on infiltrating NKG2D^+^ CD8^+^ T cells and enhances their IFNγ production. This local increase in IFNγ can then act back on the skin epithelium to enhance expression of IL-15 and expression of NKG2D ligands, leading to further recruitment/activation of effector CD8^+^ T cells. This IL-15/IFNγ feedback loop between CD8^+^ T cells and the epithelium surrounding the hair follicle is thought to be a driving force to the diseased state (7). This concept was further supported using the murine skin graft induction model, in which mice treated with IL-2/15Rβ blocking antibody were protected from disease, had significantly reduced numbers of skin-infiltrating NKG2D^+^ CD8^+^ T cells, failed to upregulate MHC molecules in the skin and presented a skin gene signature similar to an unaffected mouse (7). Overall, these findings suggest that IL-15 may contribute to ongoing disease in patients, likely by enhancing effector functions of pathogenic CD8^+^ T cells.

## JAK inhibition

JAKs phosphorylate their target substrates by transferring the terminal phosphate group from a high-energy adenosine triphosphate (ATP) molecule to the hydroxyl (-OH) residue of a tyrosine. This occurs when an ATP molecule binds to an open site within the ~300 base pair kinase domain of the active JAK. This ATP binding pocket is surrounding by the N-terminal lobe and the C-terminal lobe of the kinase domain. The N lobe helps to orient and anchor the ATP, while the C lobe binds the ATP and initiates the phospho-transfer (47). Interest in JAK inhibition as a therapeutic strategy was invigorated in 2005 when patients with myeloproliferative disorders (MPD) were found to have a JAK2 point mutation leading to its constitutively active state (48). This spurred the development of an inhibitor with activity against JAK2, and in 2011 the FDA approved the use of ruxolitinib for the treatment of patients with MPD. Shortly after, in 2012, tofacitinib was approved for patients with rheumatoid arthritis that were resistant to other treatments (49). JAKis are small membrane-permeable molecules that work by outcompeting ATP for binding in the pocket of the kinase domain, which ultimately prevents the JAK from phosphorylating its target substrate. However, due to similarities in the structure of the ATP-binding sites across the JAKs, the first generation of JAKis inhibit more than one JAK, typically in a hierarchical order based on binding site affinity. Second generation JAKis have been developed to more selectively inhibit individual JAK family members, with many currently being tested in clinical trials for a variety of autoimmune diseases (50). The preclinical rationale and the first demonstration of efficacy for JAKis to treat AA were published in 2014 (7, 51); these publications heralded the explosion in interest and clinical trials examining the efficacy and safety of JAKis for patients with AA as well as other dermatological conditions. Select clinical trials are described in **Table 2**.

## First generation JAK inhibitors

The first generation of JAKis affect more than one JAK family member and have shown promising results in the context of AA; the efficacy of first generation JAKis in AA may be due to the inhibition of multiple cytokines/JAK signaling pathways that are contributing in parallel, and are partially redundant, in disease pathogenesis. The first generation of JAKis are comprised of ruxolitinib, tofacitinib, baricitinib, and oclacitinib (52). Of this group, the first three have been explored as treatments for AA and will be discussed further.

### Ruxolitinib

Ruxolitinib is commonly regarded as a JAK1/JAK2 inhibitor (**Table 1**). The activity profile of ruxolitinib makes it an interesting therapeutic candidate for AA given how it can dampen both γ_c_ family cytokine signaling (JAK1/JAK3) and also IFNγ signaling(JAK1/JAK2). Thus, ruxolitinib is likely to act upon immune cells, which are responsive to γ_c_ family cytokine signaling, and act at the level of the hair follicle, which is responsive to IFNγ signaling. Ruxolitinib was among the first of the JAKis that demonstrated efficacy for AA. In 2014, three patients with moderate/severe AA treated with oral ruxolitinib exhibited nearly complete hair regrowth after 3-5 months of treatment. Ruxolitinib treatment in these patients resulted in fewer CD8^+^ and CD4^+^ T cells infiltrating the hair follicle and its periphery (7). The positive outcome from this initial study led to numerous investigations testing the efficacy of ruxolitinib further.

**Table 1:** JAK inhibitors with documented efficacy for treating AA.

IC_50_ (nM)				
	JAK1	JAK2	JAK3	TYK2
Tofacitinib (53)	15	71	45	472
Ruxolitinib (54)	3.3	2.8	428	19
Baricitinib (53)	0.78	2	253	14
Abrocitinib (55)	29	803	>10000	1253
Delgocitinib (56)	2.6	2.8	13	58
Upadacitinib (53)	0.76	19	224	118
Brepocitinib (57)	23	17	77	6494
Ritlecitinib (58)	>10000	>10000	33.1	>10000

IC_50_ values denote the concentration of drug that reduces enzymatic activity of each JAK member by 50%. All reported assays performed in the presence of 1 mM ATP.

From 2016 onward, case reports have appeared highlighting the use of ruxolitinib to treat AA, sometimes under special circumstances such as AA patients presenting with additional autoimmune diseases as well as in pediatric AA patients. An early case report demonstrating ruxolitinib efficacy for AA involved a patient presenting with both AA and vitiligo, a cutaneous autoimmune disease that causes depigmentation of the skin and, like AA, is mediated by CD8^+^ T cells and IFNγ. The patient received treatment for 20 weeks, and experienced significant regrowth of hair (85% of scalp coverage from a baseline 63% coverage) after only 12 weeks of treatment, and this regrowth was maintained for at least 12 weeks after treatments ceased (59). A more recent case report demonstrated one of the first uses for ruxolitinib to treat AA in a preadolescent patient. A 9-year-old patient with AT was treated twice daily with 20 mg of oral ruxolitinib, and 4 months of treatment yielded nearly complete regrowth of hair on scalp and eyebrows. The dosage was tapered to 10 mg every other day, a level that maintained efficacy with no adverse effects (60).

Early cases reports identified that hair regrowth after cessation of ruxolitinib is not sustained long-term, as patients tended to experience hair loss relapse after ending treatment. Case reports of ruxolitinib for treating AA have involved testing different treatments regimens, such as changing the duration and altering dosage, to prolong positive outcomes. A report involving two patients, one presenting with AT and one presenting with AU, were given daily ruxolitinib treatments for 13-14 months, and then tapered to less frequent dosing. The patients exhibited near complete regrowth by 6-8 months of treatment, and the tapered dosing led to maintenance of hair regrowth (61). An additional study demonstrated efficacy of lower dosing ruxolitinib to reverse hair loss in severe AA patients. Eight patients were treated twice daily with 10-25 mg of ruxolitinib for a range of 5-31 months. Interestingly, six of the enrolled patients had undergone prior tofacitinib therapy with varied results. Five of the enrolled patients attained near complete hair regrowth, with four of them having received the lower dosage of 10 mg ruxolitinib twice daily (62).

A few clinical trials testing the use of both systemic oral ruxolitinib and localized topical ruxolitinib to treat AA have been completed. A phase 2, open-label trial with oral ruxolitinib involved a cohort of twelve patients with moderate/severe AA that received daily ruxolitinib for a duration of 3-6 months. 9 of 12 patients had at least 50% regrowth of hair and continuous reduction in SALT scores over time while receiving treatment. Three months after treatment cessation, all nine responsive patients noted hair shedding, with three of those patients experiencing marked hair loss. Gene expression profiling revealed that ruxolitinib treatment normalized the aberrant IFNγ and CD8^+^ T cell-based gene signatures in the scalp, demonstrating that AA lesion biopsies taken after 12 weeks of treatment clustered closer to healthy skin biopsies than baseline AA biopsies (**Table 2**) (64).

**Table 2:** Completed or currently active clinical trials involving the use of JAK inhibitors to treat AA.

JAK inhibitor	Study type	Patient information	Dosing	Outcome	End Date	Trial ID	References
**Tofacitinib**	pilot study, open label	66 adult patients with severe AA, ophiasis, AT, or AU	5 mg twice daily for 3 months	• 42 of 66 patients were responsive. 21 of the responsive patients reached a SALT_50_ score • Greatest reduction in SALT scores observed in patients with AA (70%) and ophiasis (68%) • 20 patients were followed for 3 months after Tx cessation, and all experienced hair loss • No serious adverse effects (AE) reported	August 2015	NCT02312882 NCT02197455	Kennedy Crispin et al. (63)
**Ruxolitinib**	Phase 2, open label	12 adult patients with moderate to severe AA	20 mg twice daily for 12-24 weeks	• 9 of 12 patients were responsive, with an average of 92% regrowth • Mean SALT scores of responders: Baseline (65.8), 3 months Tx (24.8), 6 months Tx (7.3) • 3 months after cessation all 9 responders noted hair shedding, with 3 experiencing marked hair loss • No serious AE	April 2016	NCT01950780	Mackay-Wiggan et al. (64)
**Delgocitinib ointment** (LEO 124249)	Phase 2, double blind, vehicle controlled	31 adult patients with moderate to severe AA (>30% scalp involvement), randomly assigned	30 mg/g ointment applied twice daily (20) or vehicle control (11) for 12 weeks	• The primary outcome measured was change in SALT score. The mean change after 12 weeks of treatment was a decrease of 3.8 in the drug group, and a decrease of 3.4 in the vehicle group. • No serious AE	December 2016	NCT02561585	Mikhaylov et al. (65)
**Ruxolitinib cream**	Phase 2 Part A: open label Part B: double-blind, placebo controlled, followed with optional open label extension	Part A: 12 adult patients with moderate/severe AA Part B: 78 adults with moderate to severe AA randomly assigned to ruxolitinib (39) or vehicle (39) group Extension: 63 patients, 31 from ruxolitinib group and 32 from vehicle	Part A: 1.5% topical ruxolitinib cream, twice daily for 24 weeks Part B:1.5% topical ruxolitinib cream or vehicle twice daily for 24 weeks Extension: 1.5% topical ruxolitinib, twice daily for 24 weeks	Part A: 6 of 12 patients reached a SALT_50_ score (50% or greater improvement in SALT score) after 24 weeks of Tx. Part B: 5 of 39 patients in both the Ruxolitinib cream group and the placebo group reached a SALT_50_ score. Extension: 3 patients from previous ruxolitinib group reached a SALT_50_ score, and 1 patient reached a SALT_90_ score. 4 patients from previous vehicle group reached a SALT_50_ score. • Serious AE experienced by 1 patient in Part A, and 3 patients in Part B	October 2017	NCT02553330	Olsen et al. (66)
**Tofacitinib** (Xeljanz)	Phase 2, open label	12 adult patients with moderate to severe AA, AT, or AU	5 mg-10 mg twice daily for 6 months with option to extend up to 18 months.	• 11 of 12 patients showed SALT score improvement. Mean SALT score of 81.3 at baseline dropped to 40.8 at the end of treatment. • 8 of 12 patients reached a SALT_50_ score and were followed for 6 months after Tx cessation. 6 of the 8 experienced hair loss after stopping and 1 patient maintained hair regrowth during this period. • 1 patient experienced AE resulting in discontinuation of Tx	December 2017	NCT02299297	Jabbari et al. (67)
**Tofacitinib ointment**	Phase 2, open label	10 adult patients with AA (at least 2 patches), AT or AU	2% topical tofacitinib, twice daily to half of affected area for 6 months	• 3 of 10 patients were responsive, with 1 patient experiencing significant regrowth and 2 exhibiting partial regrowth. • Mean SALT score decrease of responsive patients was 34.6% • No serious AE	July 2018	NCT02812342	Liu et al. (68)
**Ritlecitinib** (PF-06651600)	Phase 2a, double blind with optional single blind extension	72 adult patients with moderate to severe AA. Randomly assigned to ritlecitinib (48) and placebo (24)	200 mg daily for 4 weeks, then 50 mg daily for 20 weeks or placebo	• At 24 weeks of Tx, 50% of patients achieved SALT_30_ scores, and 25% had achieved SALT_90_ scores by this time. • No serious AE	May 2019	NCT02974868	King et al. (69)
**Brepocitinib** (PF-06700841)	Phase 2a, double blind with optional single blind extension	70 adult patients with moderate to severe AA. Randomly assigned to brepocitinib (47) and placebo (23)	60 mg daily for 4 weeks, then 30 mg daily for 20 weeks or placebo	• At 24 weeks of Tx, 64% of patients achieved SALT_30_ scores, and 34% had achieved SALT_90_ scores by this time. • 2 patients receiving brepocitinib experienced serious AE	May 2019	NCT02974868	King et al. (69)
**ATI-501** *JAK1/JAK3 inhibitor*	Phase 2, double blind, placebo controlled	87 adult patients with AA, AU, or AT, randomly assigned	400 mg (23), 600 mg (23), 800 mg (22) or placebo (19) taken daily for 24 weeks	• The primary outcome was the percent change in SALT scores. After 24 weeks, changes were: -25.6 (400 mg), -30.4 (600 mg), -25.9 (800 mg), and -6.3 (placebo). • No serious AE	June 2019	NCT03594227	(70)
**Ritlecitinib** (PF-06651600)	Phase 2B/3, double blind, placebo controlled and dose ranging	718 adult and adolescent (> 12 years old) patients with moderate to severe AA (≥ 50% scalp hair loss)	Range of daily oral dosing, broken into a 4-week loading phase/20-week maintenance phase/24-week extension phase: •200mg/50mg/50mg •200mg/30mg/30mg •50mg/50mg/50mg •30mg/30mg/30mg •10mg/10mg/10mg •placebo	• Primary endpoint was a SALT score ≤ 20. The three highest treatment groups (31%, 22%, and 24% respectively) resulted in the most patients reaching that endpoint. • 30 mg group resulted in 14.5% of patients reaching endpoint, while the 10 mg group only had 2% of patients reach endpoint. • 12 patients, dispersed throughout test groups, experienced serious AE	December 2020	NCT03732807 **(ALLEGRO-2b/3)**	(71)
**Baricitinib** (LY3009104)	Phase 3, double blind placebo controlled	654 adult patients with severe AA. (>50% scalp involvement)	4 mg twice daily baracitinib (281), 2 mg twice daily baricitinib (184), or placebo (189)	• The primary outcome was to achieve a SALT score of 20. 38.8% of patients in the 4 mg achieved the outcome, along with 22.8% in the 2 mg group and 6.2% in the placebo group. • Serious AE reported in 13 patients, dispersed throughout all groups	January 2021	NCT03899259 **BRAVE-AA2**	King et al. (72)
**Baricitinib** (LY3009104)	Phase 2/3, double blind, placebo controlled	546 adult patients with severe AA (>50% scalp involvement), randomly assigned	4 mg twice daily baracitinib (234), 2 mg twice daily baricitinib (156), or placebo (156)	• The primary outcome was to achieve a SALT score of 20. In the 4 mg group, 35.9% of patients achieved the outcome, along with 19.4% in the 2 mg group and 3.3% in the placebo group. • Serious AE reported in 15 patients, dispersed throughout all groups	February 2021	NCT03570749 **BRAVE-AA1**	King et al. (72)
**CTP-543**	Phase 3, double blind, placebo controlled	706 adult patients with severe AA (≥ 50% scalp hair loss), randomly assigned	12 mg CTP-543, 8 mg CTP-543, or a placebo taken twice daily for 24 weeks	• The primary outcome was to achieve a SALT score ≤ 20. 42% of patients in the 12 mg group achieved the outcome, along with 30% in the 8 mg group and 1% in the placebo group. Significant differences noted as early as 8 weeks into Tx. • Serious AE reported in 9 patients dispersed throughout all groups	May 2022	NCT04518995 **THRIVE-AA1**	(74)
**CTP-543**	Phase 3, double blind, placebo controlled	517 adult patients with severe AA (≥ 50% scalp hair loss), randomly assigned	12 mg CTP-543, 8 mg CTP-543, or a placebo taken twice daily for 24 weeks	• The primary outcome was to achieve a SALT score ≤ 20. 38.3% of patients in the 12 mg group achieved the primary outcome, along with 33% of patients in the 8 mg group and 0.8% in the placebo group. • Serious AE were experienced by 5 patients in the trial.	June 2022	NCT04797650 **THRIVE-AA2**	(104)
**CTP-543**	Phase 2, double blind, placebo controlled	300 adult patients with severe AA (≥ 50% scalp hair loss), randomly assigned	Part A: 12 mg or 8 mg twice daily for 24 weeks, followed by 24 weeks of dose reduction or placebo. Part B: 12 mg or 8 mg twice daily for 24 weeks for patients experiencing loss of maintenance during Part A.	Primary outcome measures: Part A: frequency of patients exhibiting loss of maintenance upon dose reduction or drug discontinuation. Part B: frequency of patients experiencing restoration of regrowth	Est. Oct 2022	NCT04784533	-
**Ritlecitinib** (PF-06651600)	Phase 3, open label	1049 adult and adolescent (> 12 years old) patients with AA. Some patients have participated in prior PF-066511600 clinical trials.	Naïve patients: 200 mg daily for 1 month, then 50 mg daily for 35 months Previously enrolled patients: 50 mg daily for 36 months	Number of subjects reporting adverse events, abnormal vital signs, or abnormal clinical lab values.	Est. July 2024	NCT04006457 **ALLEGRO-LT**	-

A phase 2, double-blind, placebo-controlled trial examining topical ruxolitinib cream was conducted in a cohort of 16 AU patients that received twice daily ruxolitinib applications for 12 weeks. 5 of 16 patients exhibited partial regrowth in areas treated with topical ruxolitinib. However, hair loss was noted after cessation of treatments at 6-week and 12-week follow-up time points (74). Soon after, another phase 2, double-blind, vehicle-controlled trial appraised topical ruxolitinib cream in a cohort of 78 patients with moderate AA. Patients received daily applications to affected areas for a duration of 24 weeks. Both the ruxolitinib cream and vehicle treated groups resulted in 5 of 39 patients showing reduced SALT scores over the 24-week period. This study concluded that topical ruxolitinib does not significantly affect AA patients (**Table 2**) (66).

To accompany the numerous case reports and trials, the protective effects of ruxolitinib have been demonstrated using an *in vitro* hair follicle culture model. Human dermal papilla cells (hDPCs) pre-treated with ruxolitinib had significantly lower MHC Class II expression following IFNγ exposure when compared to resting hDPCs exposed to IFNγ. Furthermore, hDPCs treated with ruxolitinib showed a reversal in the expression of IFNγ-inducible genes (such as caspase-1, IL-15, IL-1β) following stimulation with IFNγ. These findings suggest that ruxolitinib plays a role in helping maintain an immune privileged state of the hair follicle, likely by dampening the downstream effects of IFNγ signaling, in addition to its effects on immune effectors (75).

Of note, a deuterated form of ruxolitinib, CTP-543, has recently been developed for the treatment of AA. Efficacy for CTP-543 has been supported by data from a phase 2, double-blind, placebo-controlled trial involving 149 adult patients with severe AA that received 4 mg, 8 mg, or 12 mg of CTP-543 twice daily or a placebo for a duration of 24 weeks. The primary endpoint was the frequency of patients achieving a SALT_50_ score, which is a 50% or greater reduction in SALT scores in comparison to baseline. The trial found that patients in the higher dosage groups (8 mg and 12 mg) had significantly more patients reach the endpoint (47% and 58%) in comparison to placebo (9%). The frequency of patients experiencing adverse effects were similar among all treatment groups, and only one patient (12 mg dosage) experienced a serious adverse effect (76). The positive outcomes from this trial led to the initiation of two larger-scale studies, THRIVE-AA1 and THRIVE-AA2, examining further efficacy and safety (**Table 2**). THRIVE-AA1 contained 706 patients receiving 12 mg CTP-543, 8 mg CTP-543 or placebo twice daily for 24 weeks, with a primary endpoint of achieving a SALT score ≤ 20. 42% of patients in the 12 mg group and 30% in the 8 mg group met this endpoint, compared to 1% of the placebo group. Additionally, 53% of patients in the 12 mg group and 42% in the 8 mg group were satisfied or very satisfied with their results after 24 weeks of treatment compared to 5% in the placebo group. Serious adverse effects were reported by 1 patient receiving 12 mg, 4 patients receiving 8 mg, and 4 patients in the placebo group (73). THRIVE-AA2 contained 517 patients randomly assigned to 12 mg CTP-543, 8 mg CTP-543, or placebo. Similar to THRIVE-AA1, the primary endpoint was to achieve a SALT score of ≤ 20. 38.3% of patients in the 12 mg group and 33% of patients in the 8 mg group met this endpoint, compared to 0.8% in the placebo group. 52% and 47% of patients in the 12 mg and 8 mg groups were satisfied or very satisfied with their results compared to 2% of patients in the placebo group (74). Together, the two THRIVE trials enrolled over 1200 patients. The positive results from these massive trials indicate that CTP-543 may be the next inhibitor granted approval for treating adult patients with AA.

### Tofacitinib

Tofacitinib is typically classified as a pan-JAK inhibitor with preference towards the JAK1/JAK3 pair (**Table 1**). The inhibitory properties of tofacitinib show it to be effective in dampening signals from the γ_c_ family cytokines (JAK1/JAK3) and IFNγ (JAK1/JAK2), indicating that it can act on immune cells and on the hair follicle. To date, tofacitinib has likely been prescribed for patients with AA more than any other JAKi.

Tofacitinib efficacy for AA was initially suggested in 2014, in which a patient presenting with both plaque psoriasis and AU exhibited complete regrowth of scalp hair after 3 months of treatment and full regrowth of all body hair after 8 months of treatment (51). Since then, many case studies have appeared supporting the efficacy of tofacitinib to treat AA. An early report involved a patient with persistent AA who received daily tofacitinib for 4 months and demonstrated near complete regrowth of hair. Transcriptional analysis of skin biopsies taken 4 weeks into treatment revealed a decrease in IFN and cytotoxic T cell gene signatures when compared to baseline biopsies. Additionally, serum levels of Cxcl10, an IFN-inducible chemokine, were noticeably lower after 4 weeks of tofacitinib treatment. However, upon ceasing treatment, the patient presented with near complete loss of hair at the 16-week timepoint (77). A more recent entry in the literature reported the efficacy of treating a patient that presented with AU, atopic dermatitis, and ulcerative colitis simultaneously. Daily tofacitinib treatments led to marked scalp hair regrowth by 4 months of treatment, along with significant improvement in itch and erythema, and colonic biopsies suggested a remissive state. The treatment dose for this patient was tapered, and, at the 10-month follow-up, all three disease states remained well controlled. This report highlights a potential advantage of pan-JAKi therapy for patients exhibiting multiple diseases of inflammatory nature (78).

There have also been ample reports promoting the efficacy of tofacitinib to treat AA in the pediatric population, a group with increased risk of developing more severe and chronic forms when compared with adults (79). Increasing the pool of safe and effective therapeutics for pediatric patients would be especially beneficial given the psychological burdens this disease carries in children (80). An initial report involved 4 patients (5-7 years of age) with AT or AU who were treated with oral tofacitinib. Drug dosing was based on concurrent clinical trials testing tofacitinib in juvenile idiopathic arthritis patients. Two AA patients demonstrated complete regrowth of hair by 3-6 months of daily treatments, another had 62% of regrowth occur, and the remaining patient exhibited no substantial regrowth (81). Shortly after, another case series was communicated detailing 11 patients (7-11 years of age) that exhibited a range of AA, from involving eyebrows only to AU. The patients received daily oral tofacitinib for at least 6 months, starting at a relatively low dose (2.5-7.5 mg daily), which was increased depending on patient tolerance and responsiveness. Nine of the patients responded well, with a mean SALT score change of 67.8%, and two remaining patients experienced no noticeable hair regrowth. The treatment appeared well tolerated, with most adverse events being characterized as mild and transient (82). These studies support that systemic tofacitinib therapy may be an effective option for treating pediatric AA.

A few clinical trials investigating the efficacy of daily oral tofacitinib to treat AA have been completed. An open-label trial was conducted with 66 patients, with the majority (71.2%) having AU at the time of enrollment. After 3 months of treatment, 32% of the patients had strongly responded by exhibiting significant improvements in SALT scores (>50% change), and the treatment was tolerated with limited adverse effects. Immunofluorescent staining of a biopsy from a strongly responding patient had a marked reduction in STAT3 expression within the follicular epithelium after 2 months of treatment, suggesting that blocking this pathway is associated with a positive outcome. 20 patients were evaluated at 3 months after cessation of treatment, and these patients had all experienced hair loss, suggesting that transient treatment with tofacitinib does not result in a durable response (**Table 2**) (63). Another open-label trial examining tofacitinib for AA involved 12 patients. Eleven patients completed the study, having received daily treatments over 6-18 months. 8 of 11 patients reached the primary efficacy endpoint of a SALT score reduction of > 50%. Upon cessation, six of the eight responding patients experienced hair loss as early as 4 weeks post treatment, while one patient presented with maintained hair regrowth even up to 6 months post treatment (**Table 2**) (67).

The use of tofacitinib as a topical therapeutic agent has also been investigated. An initial study examined the efficacy of topical 2% tofacitinib to treat three pediatric patients exhibiting patchy AA or AT. They received twice daily applications, and 2 of 3 patients (one with patchy AA and the other with AT) experienced 80% and 95% regrowth of scalp hair. However, a third patient with AT failed to exhibit any regrowth (83). Shortly after, a clinical trial was completed which tested the efficacy of daily application of 2% tofacitinib ointment in ten adult patients with moderate/severe AA (≥ 2 patches of scalp hair loss). 3 of 10 patients had hair regrowth occur, with an average SALT score change of 34.6% and minimal adverse effects (**Table 2**) (68). Larger, placebo-controlled studies are needed to confirm the benefits for topical delivery of tofacitinib, and, although the data above appear to show an efficacy signal, improved topical delivery methods will likely be required. Overall, however, there is substantial support for the use of systemic tofacitinib as a therapeutic option for patients with AA.

### Baricitinib

Baricitinib is considered a dual JAK1/JAK2 inhibitor given the similarly strong binding interactions with those two proteins (**Table 1**). Efficacy of baricitinib for AA was initially suggested in 2015, when a CANDLE patient was being treated with this drug; the patient exhibited comorbid AA and demonstrated complete hair regrowth after 9 months of treatment (84). This clinical data was further supported by studies done in the murine model of AA in the same report. Systemic administration of baricitinib led to reduced frequency of mice developing disease, reduction of CD8^+^ T cell infiltrates and a reduction of MHC Class I and Class II expression in the skin, suggesting baricitinib may help prevent the collapse of an immune privileged state of the hair follicle. Additionally, topical baricitinib applied onto lesions of AA mice led to nearly complete regrowth of hair (84). A later published case-report further indicated efficacy for baricitinib in the treatment of AA. This report involved an adult patient with progressive AT who received daily oral baricitinib. She was initially unresponsive to a starting dose of 2 mg; however, upon increasing the dosage to 4 mg, she experienced remarkable hair regrowth. 8 months of treatment resulted in 97% of regrowth on the scalp as well as regrowth on eyebrows and eyelashes. After 13 months of treatment, regrowth had been maintained, and no adverse effects were noted (85).

In the past few years, larger clinical trials testing baricitinib to treat AA have been completed. A phase 2, double blind, placebo-controlled trial randomized 110 patients with severe AA into four test groups: placebo, 1 mg, 2 mg, or 4 mg of baricitinib daily. After 12 weeks of treatments, interim SALT scores were assessed, and the 2 mg and 4 mg groups presented with the highest frequency of patients achieving SALT_30_ scores (29.6% and 33.3% respectively) (86). These two higher treatment groups were selected for continued usage, with patients initially assigned to the 1 mg dosing being transitioned to a 4 mg dosage for the remainder of the trial. A later interim analysis at 36 weeks of treatment aimed to identify the frequency of patients with SALT scores ≤ 20. The 2 mg and 4 mg groups had significantly higher frequencies of patients, 33% and 51.9% respectively, meet the criteria in comparison to the placebo group(3.6%) (**Table 2**). Overall, this study demonstrated that longer durations of treatment with higher dosages of baricitinib were effective at stimulating scalp hair regrowth and was well tolerated, with most adverse effects being classified as mild (86).

The positive results from the previous trial spurred the formation of two large-scale phase 3 trials looking at 2 mg or 4 mg of oral baricitinib to treat severe AA. The two trials, termed BRAVE-AA1, and BRAVE-AA2 contained 654 and 546 patients, respectively. Each trial randomly assigned patients to daily treatments consisting of placebo, 2 mg, or 4 mg of baricitinib, with a primary outcome of achieving a SALT score of ≤ 20. After 36 weeks of treatment, the 4 mg group had 35.9-38.8% of patients achieve the primary outcome, while the 2mg group had 19.4-22.8% and the placebo had only 3.3-6.2% of patients reach the primary outcome (**Table 2**) (72). Favorable results from the BRAVE-AA1 and BRAVE-AA2 trials led to the first-in-disease systemic FDA-approved treatment for AA. On June 13^th^ 2022, the FDA approved a once-daily regimen of baricitinib (Olumiant) available as 1 mg, 2 mg, or 4 mg tablets for adults with severe AA (87). Approved FDA labeling of Olumiant includes boxed warnings for potential serious adverse effects. This moment marks an important time in history for AA patients and their clinicians alike who now have a more accessible treatment option available. This is especially crucial given how insurance approval has historically been an issue for AA patients seeking off-label usage of JAKis (88).

## Second generation JAK inhibitors

Second generation JAKis were designed to specifically inhibit a single JAK family isoform, which may allow for more selective intervention of inflammatory diseases based on the contributing cytokines and their respective JAK signaling molecules. In addition, their more limited range of inhibitory effects may reduce the potential for adverse events. Despite their relatively recent development, studies have already begun to emerge describing efficacy for the resolution of hair loss in AA patients. The results from select studies will be discussed further below.

### Abrocitinib

Abrocitinib (PF-04965842) is a JAK1-specific inhibitor that has shown efficacy in treating patients with atopic dermatitis (AD), which is a disease marked by a higher risk of developing AA (**Table 1**) (89). A 2022 case report demonstrated efficacy of abrocitinib for AA, when a patient presenting with both AD and AU began a daily oral regimen of 200 mg. Within 12 weeks, patchy hair regrowth was noted, and full regrowth of scalp hair was observed after one year of treatment. The patient’s AD lesions were noticeably improved as well, suggesting that this selective JAKi may be a promising option for patients with dual AD and AA diagnoses (90). Another recent report highlighted two AD patients presenting with severe AA that had noticeable hair-regrowth occur upon extended duration of abrocitinib therapy. Both patients were enrolled into a long-term clinical trial termed JADE EXTEND after showing responsiveness to abrocitinib, noted by improvement in AD, in an initial shorter treatment window. The patients had noticeable hair regrowth occur at 12-14 weeks into the trial that maintained throughout the course of treatment (91).

### Upadacitinib

Upadacitinib (ABT-494) is a JAK1-specific inhibitor which was initially shown to be effective in treating AD (**Table 1**) (92). A case report describing two patients with AD and comorbid AA revealed potential efficacy of upadacitinib for treating AA. One patient had AU (SALT score 100), while the other patient had a resistant patch of AA (SALT score 43). Near complete hair regrowth was observed for both patients after 4 months of treatment with 30 mg daily (93). A more recent case report again indicated efficacy in treating a patient presenting with dual AD and AA (SALT score 89.2). The patient had failed multiple therapies for both diseases, but upon switching to 30mg of daily upadacitinib, the patient demonstrated remarkable clinical improvements in both AD and AA, with no reported adverse effects. Larger, placebo-controlled studies will be needed to determine true efficacy in patients with AA (94).

### Brepocitinib

Brepocitinib (PF-06700841) is an inhibitor of JAK1 and TYK2 that was initially shown to be effective in treating patients with plaque psoriasis (**Table 1**) (95). Efficacy for brepocitinib for treating AA was demonstrated in the recently reported ALLEGRO trial, where it was tested in parallel with ritlecitinib, which is discussed further below. 47 patients with severe AA were treated daily with 60 mg of oral brepocitinib for 4 weeks, and then tapered to a 30 mg daily dose for 20 additional weeks. After 24 weeks of treatment, 64% of patients achieved the primary outcome of a SALT_30_ score, which is a 30% or greater reduction in SALT scoring in comparison to baseline. Two patients discontinued use after experiencing serious adverse effects (69). Lesional biopsies taken from patients receiving brepocitinib (or ritlecitinib) at the 12- and 24-week timepoints revealed significant decreases in CD3^+^ and CD8^+^ T cell counts in comparison to baseline levels. Transcriptomic analysis from biopsies also showed a significant decrease in inflammatory gene signatures (immune genes, Th1, Th2 and IL-12/IL-23) in the patients receiving either brepocitinib or ritlecitinib when compared to placebo (**Table 2**) (96). The positive outcome from this initial trial warrant further investigations into the efficacy of brepocitinib for treating AA, although it appears that further investigation and development by the manufacturer have focused on ritlecitinib over brepocitinib for the treatment of AA.

### Ritlecitinib

Ritlecitinib (PF-06651600) is an inhibitor specific for JAK3 and the tyrosine kinase expressed in hepatocellular carcinoma (TEC) family of kinases (**Table 1**) (97). It acts through irreversible, covalent interactions with the cysteine 909 residue found on the active site of these kinases. The remaining members of the JAK family all possess a serine residue at this site, which accounts for the selectivity of ritlecitinib for JAK3 (58). This feature is likely clinically relevant given the use of JAK1, JAK2, and TYK2 broadly by wide-ranging receptors responsible for blood and tissue homeostasis and protective responses against pathogens. Additionally, targeting JAK3 spares signaling of immunoregulatory cytokines such as IL-10R (TYK2/JAK1), IL-27R (JAK1/JAK2), and IL-35R (JAK1/JAK2) (98), whose activity may contribute to the prevention of autoimmunity to the hair follicle, potentially making a selective JAK3 inhibitor a more potent strategy than pan-JAK inhibition. As previously mentioned, JAK3 associates exclusively with receptors for γ_c_ cytokines; because downstream signaling from γ_c_ receptors are most often transmitted in cells of the immune system, ritlecitinib represents a JAK inhibitor that may act specifically through the modulation of the immune compartment without directly influencing the hair follicle itself.

Ritlecitinib is the only irreversible JAKi being assessed for clinical use for AA patients. Efficacy of ritlecitinib for AA was demonstrated during a recently reported clinical trial (phase 2, double-blind, placebo-controlled) termed ALLEGRO, where a primary outcome was designated as a 30% improvement in SALT score. 48 patients with severe AA (≥ 50% hair loss on scalp) were treated with a loading regimen of 200 mg daily of ritlecitinib for 4 weeks, and then treated with 50 mg daily for 20 weeks. By the 24^th^ week of treatment, 50% of patients receiving ritlecitinib had reached the primary outcome, while only 3% of patients receiving placebo reached that endpoint. The number of patients experiencing adverse effects were similar between the groups; overall ritlecitinib was generally well tolerated by patients and may be an effective agent for restoring hair growth in patients with severe AA (69). Phase 3 and long-term extension trials are underway for ritlecitinib in patients with AA (**Table 2**).

## Side effects of JAK inhibitors

While JAKis have demonstrated great promise for reversing the immune-mediated hair loss in AA, they also present a risk for potentially severe side effects. This notion is not necessarily surprising given the redundancy of JAK proteins for cytokine, growth factor, and hormone receptor signaling. Commonly documented minor adverse events experienced by AA patients taking JAKis include acne, headache, nausea, urinary tract infections, respiratory tract infections, anemia, thrombocytopenia, neutropenia, and elevated creatinine levels (99). Patients using JAKis also commonly exhibit elevated LDL levels, which is a known risk factor for cardiovascular disease (99, 100). Serious adverse effects most often reported in AA trial patients receiving JAKis include varicella zoster emergence, pneumonia, tuberculosis, sepsis, and the development of non-melanoma skin cancer (99).

Beyond AA, more extensive safety analyses have been reported for patients using JAKis to treat other diseases such as rheumatoid arthritis, atopic dermatitis, psoriatic arthritis, ulcerative colitis, and myeloproliferative disorders. Results from these studies should be carefully considered until more large-scale safety trials are completed specific for the AA population. A recent meta-analysis comprised of over 126,000 JAKi patient cases for the above-mentioned diseases found that the most common adverse effects experienced were infections (herpes, influenza, fungal, and mycobacterial), thrombosis and pulmonary embolism, and neoplasms (101).

The FDA has issued boxed warnings to all JAK inhibitors that are being used for inflammatory diseases. These cautions constitute the highest level of safety warning assigned by the FDA, and are intended to alert consumers of major potential risks associated with the drug (102). In 2020, a large-scale phase-4 safety study compared the side effects of RA patients receiving 5-10 mg twice daily oral tofacitinib to patients receiving TNFα inhibitor injections for a mean duration of about 40 months. This robust study contained more than 1450 patients in each treatment group, and primary endpoints were to assess rate of cancers (excluding nonmelanoma skin cancer) and major adverse cardiovascular events (MACE) including death from cardiovascular causes, nonfatal stroke, and nonfatal myocardial infarction. A goal of this study was to examine whether tofacitinib met noninferiority criteria for the two primary endpoints; meeting this criterion indicates that there is no difference between the treatments based on the boundaries of the confidence intervals associated with the hazard ratio (103).

The incidence of cancers reported during follow-up appointments (median of 4 years), was 4.2% for tofacitinib users and 2.9% for TNFα users, with a hazard ratio of 1.48 (tofacitinib:TNFα). The most common cancer experienced among tofacitinib users was lung cancer, while breast cancer was most common among patients receiving the TNFα inhibitor. The frequency of tofacitinib users experiencing MACE was 3.4% compared to 2.5% of patients receiving TNFα inhibitor, with a hazard ratio of 1.33 (tofacitinib:TNFα). The most common MACE experienced was nonfatal myocardial infarction among tofacitinib users and nonfatal stroke among patients receiving the TNFα inhibitor. When compared to TNFα inhibitors, tofacitinib failed to meet noninferiority criteria for both incidence of cancers and MACE (103).The study determined that there is indeed a difference in adverse events experienced between users of tofacitinib and TNFα inhibitors. Of note, the efficacy was similar among all patients regardless of treatment, suggesting that in the context of RA, tofacitinib performs similarly to a well-documented biologic, but also presents a significantly greater safety risk (103).

Overall, JAKis have potential to cause off-target harm in addition to their therapeutic benefit. Thus, usage of JAKis should only be considered once the risks are clearly communicated to and comprehended by patients. Nevertheless, as more large-scale clinical trials are completed, more FDA approvals of JAKis to treat AA are likely in the not-too-distant future. 

## Conclusion

The past decade has been an exciting time for the identification and testing of new therapeutics for patients with AA. The therapeutic pool is changing from use of broad-acting immunosuppressants to more inferred and targeted approaches. These advances are possible due to the identification of novel disease mechanisms in basic science laboratories and pre-clinical AA animal models. Recent therapeutic approaches are targeting the actions of pro-inflammatory cytokines, which have been identified as crucial players and determinants of the onset, progression, and severity of AA. JAKis have taken the spotlight for now. However, despite the recent FDA approval of the first JAKi to treat AA, side effects and incomplete efficacy for refractory patients begs the question, “What else is there?” As more mechanisms of this enigmatic disease are unraveled, the arsenal of therapeutic options for AA will surely continue to grow.

## Author contributions

Both authors were responsible for writing and revising the manuscript.

## Funding

This work was supported by the Department of Veterans Affairs (VA Merit Award I01 BX004907 to AJ), the National Institutes of Health (R01 AR077194 and K08 AR069111 to AJ), and the University of Iowa Department of Dermatology.

## Conflict of interest

AJ has served as a consultant for and has received institutional payments for study-related costs from Pfizer.

The remaining author declares that the research was conducted in the absence of any commercial or financial relationships that could be construed as a potential conflict of interest.

## Publisher’s note

All claims expressed in this article are solely those of the authors and do not necessarily represent those of their affiliated organizations, or those of the publisher, the editors and the reviewers. Any product that may be evaluated in this article, or claim that may be made by its manufacturer, is not guaranteed or endorsed by the publisher.
